# Comparative Evaluation of Mechanical Properties and Bond Strength of Glass Ionomer Cement reinforced with three different concentrations of Silver Nanoparticles as against Conventional Glass Ionomer Cement in Primary Teeth.

**DOI:** 10.12688/f1000research.133455.1

**Published:** 2023-09-01

**Authors:** Harikishan Kanani, Monika Khubchandani

**Affiliations:** 1 Pediatrics and Preventive Dentistry,, Sharad Pawar Dental College and Hospital, Datta Meghe Institute of Higher Education and Research, Wardha, Maharashtra, 442001, India

**Keywords:** Restorative material, Glass ionomer cement, silver nano particles.

## Abstract

Introduction:

Restorative dental materials are defined as substances that are used to repair, replace, or enhance a patient’s teeth. Various materials used in paediatric dentistry are zinc oxide eugenol, glass ionomer cement, resin composite, calcium hydroxide, silver amalgam, giomers etc. GIC is a biocompatible material having a low thermal expansion coefficient and fluoride release property. There are still a few drawbacks of GICdue of their poor mechanical properties therefore addition of Silver Nanoparticles demonstrated improved mechanical and bactericidal capabilities. There hasn’t been much study on the quality of the bond contact between silver nanoparticles and dentin, as well as the color’s durability.

**Aim:** To evaluate and compare the mechanical properties and bond strength of Glass ionomer cement reinforced with three different concentrations of silver nanoparticle as against conventional glass ionomer cement in primary Teeth.

**Materials and Method**:

Silver nanoparticles will be prepared using three chemicals namely, silver nitrate, sodium citrate and tannic acid. Traditional GIC (GC Fuji II, GC Corporation, Tokyo, Japan) will be purchased. Three different concentrations of silver nanoparticles will be prepared i.e., 0.2, 0.4, and 0.6%. The GIC specimens will then be divided into 4 groups: GIC without silver nanoparticles (AgNPs),0.2%,0.4% and 0.6% AgNPs. Mechanical properties will be checked such as compressive, tensile, and bond strength using Universal Testing Machine.

Expected Results:

GIC reinforced with silver nano particles is expected to have better mechanical strength, less microleakage and wear resistance, greater fracture resistance and adhesive bond strength.

Conclusion:

GIC reinforced with silver nano particles will be expected to have better mechanical and physical properties than conventional Type II GICs and is expected to be a promising material for restoration in primary teeth.

## Introduction

Restorative dental materials are defined as substances that are mostly used to repair, replace, or enhance a patient’s carious teeth. Dental caries experienced a sharp drop around the turn of the 20th century, and dental enhancement and health became more popular. ^1^ Properties of ideal restorative materials would be: fracture resistance, compressive strength, tensile strength, no microleakage, hardness, adhesion, higher bond strength, ease of clinical use, good clinical performance, economical, bio-compatible and cost-effectiveness. ^2^ However, the appropriate restorative material that meets all the properties has yet to be formed. ^3^

The glass ionomer cement (GIC) was invented by Wilson & Kent (1972). ^4^ This material offers plenty of applications in paediatric dentistry such as restorations, core build-up, pit and fissure sealants, liners and bases, luting materials, and orthodontic bracket adhesives. GIC is a biocompatible material with a low coefficient of thermal expansion and fluoride releasing property. ^5^

GIC still has significant downsides, though, because of its poor mechanical qualities, which include low resistance to fracture, poor resistance to wear, and formal disintegration on water sorption. ^6^ Secondary caries and restoration failures are caused by these mechanical defects, which encourage the formation of bacterial colonies, especially Streptococcus mutans. ^7^ Fluoride release underlies the majority of GIC’s antibacterial activity. ^8^ To boost antibacterial and mechanical qualities without compromising their bond strength and reduce the development of secondary caries, fillers including silicates and fluorides were added to GIC. ^9^

Nanotechnology is the study of making high-quality structures and materials made of particle substances with sizes ranging from 1–100 nm. ^10^ Among all the materials used in nanotechnology silver has been employed in its ionised forms as nanoparticles. A metal reinforced GIC that is stronger and tougher was previously made with the use of silver alloy powder. ^11^ How effective silver or its compounds are against bacteria, viruses and fungus is determined by the silver ions amount created and their ability to interact with microorganism cell membranes. ^12^

Silver powder is sintered to glass after being heated to a high temperature. Silver-sintered powder has the potential to increase abrasion resistance and durability. ^12^

When contrasted, Dentin’s mechanical attributes (compressive strength of 297 MPa, tensile strength of 44.4 MPa, and hardness of 52 to 64 KHN) outweigh those of GIC (fracture toughness of 0.72 MPa m1/2, compressive strength of 196-251 MPa, tensile strength of 18 to 26 MPa, and hardness of 87 to 177 KHN). ^12^ According to previous study, tiny silver nanoparticles may occupy the crevices between the bigger glass particles and give an additional bonding site for the polyacrylic polymer, reducing the possibility of the cement dissolution. ^5^ As when silver nanopowder were incorporated into GIC it is revealed that when the concentration of silver nanopowder raised, bacterial colonies are reduced. ^5^ Higher concentration of silver nanoparticles showed a significant improvement in mechanical properties in relation to dentin.

There has not been enough research on the strength of the binding contact between dentin and silver nanoparticles, as well as the colour stability. Hence, objective of this study is to assess and compare mechanical characteristics, colour stability, and bond strength of primary teeth filled with GIC reinforced with various concentration of silver nanoparticles.

### Objective

In this study we aim to evaluate and compare the mechanical properties of GIC reinforced with three different concentrations of silver nanoparticles as against traditional GIC. ^16^

***Trial design:*** An *in vitro* study.

**Study setting**

This is an *in vitro* study and will be conducted at the Department of Paediatric and Preventive Dentistry, Sharad Pawar Dental College in collaboration with Research and development house, DMIHER, Sawangi (Meghe), Wardha.

### Eligibility criteria

*Inclusion criteria*

• Silver nanoparticle liquid (AgNPs) <100 nm particle size.
• Conventional GIC (GC Fuji II, GC Corporation, Tokyo, Japan).
• Primary molars close to their shedding time, affected by gross caries or the tooth that are over-retained will be extracted and included in this study.

*Exclusion criteria*

• Extracted tooth with enamel cracks, fracture, malformation, erosion, restoration will be excluded in the study.
• Permanent teeth shall not be taken into consideration.
• Primary anterior teeth will be excluded from the study.

## Methods

*Materials used*

Traditional GIC (GC Fuji II, GC Corporation, Tokyo, Japan) shall be procured and

Three chemicals:

A. silver nitrate
B. sodium citrate
C. tannic acid

*Synthesis of silver nanoparticles*

Silver nitrate, sodium citrate, and tannic acid will be used to prepare silver nanoparticles for this *in vitro* investigation. Three different concentrations of silver nanoparticles 0.2, 0.4, and 0.6% will be synthesized by chemical reduction of reducing agents i.e. sodium citrate is used for reduction of silver ions (Ag+) in aqueous or non-aqueous solutions of silver nitrate. This reducing chemical cause the reduction of Ag+ to metallic silver (Ag0), which then aggregates into oligomeric clusters. In the end, these clusters result in the emergence of metallic colloidal silver particles.

Following that, the GIC samples will be split into four groups:

GROUP 1: GIC without silver nanoparticles (AgNPs) (n=26)

GROUP 2: GIC with 0.2% silver nanoparticles (AgNPs) (n=26)

GROUP 3: GIC with 0.4% silver nanoparticles (AgNPs) (n=26)

GROUP 4: GIC with 0.6% silver nanoparticles (AgNPs) (n=26)

The extracted teeth will be collected from an operating room and dental clinic. The teeth will be carefully examined, which will be included in the criteria. For 1 month, the teeth will be stored in a 0.1% thymol solution with 0.9% isotonic sodium chloride (5°C) until the beginning of the experiment. We will use a diamond bur at a slow-speed handpiece with continuous water cooling, perpendicular to the tooth’s long axis, and cavity preparation will be done from approx 2.0 mm of the tissue along with the cusps without exposing the pulp. The 104 teeth will be categorized into four groups with an equal distribution, which will include group 1 (applying GIC on dentin as the control group), group 2 (applying GIC with silver nanoparticles (AgNPs) (0.2%) on dentin), group 3 (applying GIC with silver nanoparticles (AgNPs) (0.4%) on dentin), and group 4 (applying GIC with silver nanoparticles (AgNPs) (0.6%) on dentin). For the preparation of the control group, the ratio of powder and liquid will be taken as per the manufacturers’ instructions, and they will be mixed on a glossy paper pad.

Then, utilising a universal testing equipment (Instron universal testing machine), mechanical qualities including compressive strength, tensile strength, and bond strength will be examined. Utilising universal hardness testing equipment, the hardness will be evaluated.

### Expected outcomes

In comparison to standard Type II GIC, GIC reinforced with silver nanoparticles is anticipated to have improved mechanical and physical qualities, less microleakage, and fewer risks of secondary caries due to the antibacterial ability of silver nanoparticles. A potential restorative material for primary teeth is GIC enhanced with silver nanoparticles.

### Sample size

Sample size is determined using the following formula,

$$\begin{array}{c} n=\left(Z\;1-\alpha /2+Z\;1-\beta \right)2\;p1\left(1-p1\right)+p2\left(1-p2\right)\;\\ \left(p1-p2\right)2 \end{array}$$

Proportion of outcome (p1) = 0.58

Proportion of outcome (p2) = 0.65

Level of significance (α) = 0.05

Power (1- β) = 0.80

Z alpha value = 1.96

Z beta value = 0.85

Input: Effect size f = 0.34

α error probability = 0.05

Power (1-β error probability) = 0.85

Numerator df = 10

Number of covariates = 1

Output: Noncentrality parameter λ = 25.0000000

Critical F = 1.759

Denominator df = 76

Total sample size = 104

Actual power = 0.9125

Sample size = 104

Total Sample size = 104

As per Wassefy *et al*. ^13^

### Statistical method

All the results will be calculated using SPSS version 27 software (IBM Corp, 2020) (RRID:SCR_002865). Data for outcome variables will be tested for normality using kalmogorov-smirlov. Comparative analysis over the outcome of functional occlusion in different malocclusion will be evaluated and measurement of depth of curve of Spee and Wilson in millimetres respectively. ANOVA will be used to find significant difference in mean in comparison of four groups. The Tukey test will be used for comparative evaluation of measurement in between two groups pairwise. P-value≤0.05 will be considered significant with a 5% level of significance and 95% confidence of interval.

### Dissemination

Article will be published in indexed journal/findings will be shared in conferences.

### Study status

The study is not yet started. However, we are planning to begin the research in August 2023 and complete in around six months.

## Discussion

Anti-bio adhesion coatings, antibiotic coatings, and silver inclusion are some of the ways proposed to inhibit bacterial attachment to bare material surfaces; the latter has attracted the curiosity of many researchers.

Noha A. El-Wassefy *et al.,* investigated adding silver nanoparticles to GIC. This showed that this can prevent the establishment of S. Aureus biofilms while having just a minor impact on mechanical properties. ^13^

Faisal Mohammed Abed *et al.*, conducted an in vitro investigation and found that GIC added with AgNPs positively affected the bond quality in dentin interaction at concentrations more than 0.4%. ^5^

A broad-spectrum antibiofilm effect of silver has been found, with little to no bacterial resistance. According to literature on the antibiofilm actions of silver compounds, silver can be used in bone cement and wound dressings. ^14^^,^^15^ Thus, data from past studies indicate the advantages of inserting silver nanoparticles, which show increased mechanical and antibacterial properties. There is still not enough research on the strength of the bond contact between dentin and silver nanoparticles as well as durability of colour. As a result, the goal of this work is to evaluate and compare the mechanical properties, colour stability, and bond strength of primary teeth filled with glass ionomer cement at varying concentrations of silver nanoparticles.

### Ethical considerations

Ethical approval received from Datta Meghe Institute of Higher Education and Research, Sawangi, Wardha

IEC reference number - DMIHER (DU)/IEC/2023/566

## Data Availability

Zenodo: Spirit _checklist Harry, https://doi.org/10.5281/zenodo.7788338. ^16^ Data are available under the terms of the Creative Commons Attribution 4.0 International license (CC-BY 4.0).
