# Supplementary material for: Comparative Evaluation of Mechanical Properties and Bond Strength of Glass Ionomer Cement reinforced with three different concentrations of Silver Nanoparticles as against Conventional Glass Ionomer Cement in Primary Teeth
Source: F1000Res. 2024 Sep 26;12:1084. Originally published 2023 Sep 1. [Version 2] doi: 10.12688/f1000research.133455.2 (PMC12265879; doi:10.12688/f1000research.133455.2)
Supplement: Peer review history

# Reviewer report: Reviewer response for version 2

**DOI:** 10.5256/f1000research.172182.r379209

**Published:** 2025-04-30

**Authors:** Muna Saleem Khalaf

**Affiliations:** 1 Pedodontics and Preventive Dentistry, University of Baghdad, Baghdad, Iraq

Title:

 The title is too detailed and long. Review changing it to “Impact of Silver Nanoparticle Inclusion to Reinforced Glass Ionomer Cement on Mechanical Properties and Bond

 Strength in Primary Teeth"

 Introduction:

- The introduction requires a few corrections or additions. For example, defining restorative dental materials as to replace carious teeth should be corrected. Restorative dental materials replace lost dental tissue or structure that maybe due to caries in addition to many other causes such as trauma, wear…. etc. The drawbacks of GIC should concentrate on the aim of this study to enhance these downsides. Mentioning overall drawbacks or weaknesses of the material was fine but to focus on the weak bond strength of the material to dentin and enamel and other mechanical properties to be studied is more important to justify the need for this study.
- Silver Nanoparticles have shown antibacterial effect and remineralization of dental tissues. Their preventive role in reducing caries progression and increasing hardness of dental tissue has not been mentioned. More reference on the effect of Silver Nanoparticles in increasing hardness and remineralization on bonding strength should be considered.
- At the end of the introduction, the objectives of the study included color stability while it was not mentioned in the aims. Was this measured? If color stability was measured, the method of measurements was not mentioned. Details should be provided on the method and device or exclude it from the study.

Aim/Objective:

- Be more detailing when writing the aims of the study. Mention the mechanical properties specifically to be included in the study.

Materials and Methods:

- This part needs more details on the materials used and their production, method of preparation (especially Silver Nanoparticles in different concentrations) and manipulation. Fuji II is provided in the form of liquid and powder. Silver Nanoparticles at different concentrations are used but the form of addition is not clarified. Added as powder by weight to the powder of Fuji II? Or to the liquid constituent. Has their addition been taken in account to the overall volume of the GIC material. Meaning: if added to the powder or liquid it will increase the amount and will this affect the mixing property or the setting property of the material? Was the mixing of material manually or by a mixing device? This should be clarified because the method of mixing has a significant effect on the properties of the material. How was the material applied in the cavity?
- Sample collection and preparation:
- The collection of teeth needs to be more precise. Was a consent obtained from the child, parent or guardian? The criteria for collection, centers or clinics whether public or private. preparation of teeth also needs to be clarified such as cleaning and polishing before storage.
- Criteria for cavity preparation should be mentioned. Standardization involving drilling device, type of bur, depth of cavity. 2,0 mm of the tissue along with the cusps is unclear. Provide precise dimensions. What was the severity of caries in carious teeth. How will the severity be recorded? Unify the sample; carious teeth or sound teeth at shedding time. By what criteria or index was the depth or cavity preparation decision considered enough. The reach of sound dentin? Affected dentin? Within a standard thickness of dentin? If the cavity was finished, confirmation of sound or affected dentin was obtained by which mean? A device or dye?

Discussion:

- The writing of the discussion section should focus on the objectives of the study. The effect of incorporating Silver Nanoparticles in GIC on bacterial establishment and antibiofilm effect has not been included as an aim of this study. Therefore, should not be discussed. This also applies for the use of silver compounds in wound dressing and bone cement. Focus on the tests carried out. Rewrite the discussion to avoid being presented as review of literature.

Is the study design appropriate for the research question?

Partly

Is the rationale for, and objectives of, the study clearly described?

Partly

Are sufficient details of the methods provided to allow replication by others?

Partly

Are the datasets clearly presented in a useable and accessible format?

Partly

Reviewer Expertise:

dentistry, pedodontics, child behavior

I confirm that I have read this submission and believe that I have an appropriate level of expertise to confirm that it is of an acceptable scientific standard, however I have significant reservations, as outlined above.

# Response

**Published:** 2025-05-16

**Authors:** Harikishan Kanani

**Affiliations:** Pediatrics and Preventive Dent, Sharad Pawar Dental College and Hospital, Datta Meghe Institute of Higher Education and Research, Wardha, Maharashtra, India

Thank you so much

# Reviewer report: Reviewer response for version 2

**DOI:** 10.5256/f1000research.172182.r379207

**Published:** 2025-04-23

**Authors:** Paulo J. Palma

**Affiliations:** 1 University of Coimbra, Coimbra, Portugal

The study aims to evaluate the effect of silver nanoparticle (AgNP) reinforcement on the mechanical properties and bond strength of glass ionomer cement (GIC) in primary teeth. This is a relevant topic, as improving the properties of GIC could lead to better clinical outcomes in pediatric dentistry. However, several areas need to be addressed to enhance the manuscript's quality and suitability for a high-impact journal.

 Specific Comments:

 Title:

- The title is quite long. Consider shortening it for clarity and impact. For example: "Silver Nanoparticle-Reinforced Glass Ionomer Cement Improves Mechanical Properties and Bond Strength in Primary Teeth"

Abstract:

- The abstract should be concise and clearly state the study's purpose, methods, key findings (if available), and conclusion.
- The current abstract lacks specific details about the methodology (e.g., how the teeth were prepared, the exact mechanical tests used) and doesn't include any quantitative results.
- Suggestion: Revise the abstract to include more specific information.

Introduction:

- The introduction provides a good overview of the background and rationale for the study.
- The literature review is generally adequate, but could benefit from more recent and high-impact publications.(e.g., Mansoor A, et al., 2025 (Ref 1); Lehmann A, et al., 2024 (Ref 2); Omer et al 2024).
- A stronger connection between the identified problems of GIC and how AgNPs specifically address those problems would improve the flow. For instance, more emphasis on the specific mechanisms by which AgNPs enhance mechanical properties and reduce bacterial activity. It is suggested to add a sentence or two explaining how AgNPs improve mechanical properties (e.g., 'AgNPs fill the gaps in the GIC matrix, increasing its density and strength.') and reduce bacterial activity (e.g., 'AgNPs release silver ions, which have a broad-spectrum antibacterial effect.').
- The last paragraph of the introduction mentions color stability but this is not mentioned in the aim or methods. If it is to be studied, it needs to be formally included.
- Suggestions:

- Include more recent references.
- Elaborate on the mechanisms of AgNP action.
- Ensure all aspects mentioned in the introduction (e.g., color stability) are included in the aim, methods, results, and discussion. It is suggested to add a sentence to the Aim/Objective section: 'To also evaluate the color stability of GIC reinforced with different concentrations of silver nanoparticles.' If color stability is included, add details to the Methods section on how color stability will be measured (e.g., using a spectrophotometer). If color stability will not be measured, remove the reference to it from the introduction.

Aim/Objective:

- The aim is clearly stated. However, it could be more concise.
- The aim mentions mechanical properties. The methods mention hardness, compressive strength, tensile strength, and bond strength. Please ensure that the aim includes all of these.
- Suggestion: Ensure that the aim is in line with what is measured in the methods.

Materials and Methods:

- This section needs significant improvement in terms of detail and clarity.
- Study design: The study design is described as "An in vitro study." This should be more detailed.
- Sample Size Justification: The sample size calculation is present, but it's unclear why two different calculations are shown. The text refers to Wassefy et al., but it is unclear if this study is the primary source for the power calculation. Provide a clear justification for the final sample size of 104.
- Materials:
- Provide more specific information about the GIC (GC Fuji II). Include the manufacturer's details (city, country). (GC Fuji II, GC Corporation, Tokyo, Japan)
- Provide the manufacturer and grade/purity of the chemicals used: silver nitrate, sodium citrate, and tannic acid. Silver nitrate (Sigma-Aldrich, St. Louis, MO, USA, 99.99% purity), sodium citrate (Sigma-Aldrich, 99% purity), and tannic acid (Sigma-Aldrich, reagent grade) will be used.
- Synthesis of Silver Nanoparticles:

- This section lacks critical details. The method for synthesizing AgNPs must be described in sufficient detail to allow for replication. Include:

- Exact concentrations of the starting materials (silver nitrate, sodium citrate, and tannic acid).
- The order of addition of the reagents.
- Reaction temperature and time.
- Stirring speed.
- pH of the solutions.
- Any purification steps (e.g., washing, centrifugation).
- Characterization methods used to confirm the size and morphology of the synthesized AgNPs (e.g., TEM, DLS). This is crucial.

- It is not sufficient to say "redução química de agentes redutores". Silver nanoparticles were synthesized using a previously described method [Reference]. Briefly, 100 ml of 1 mM silver nitrate solution was heated to 90°C under constant stirring. Then, 10 ml of 1% sodium citrate solution was added rapidly, and the solution was stirred for 30 minutes until a color change occurred. The solution was then cooled to room temperature. The pH was not adjusted. The resulting AgNP solution was washed three times by centrifugation at 10,000 rpm for 15 minutes to remove excess reactants. The size and morphology of the synthesized AgNPs were characterized using Transmission Electron Microscopy (TEM) and Dynamic Light Scattering (DLS).

- Specimen Preparation:

- The preparation of the extracted teeth needs more detail.
- Specify the number of teeth per group (it says n=26 in one section and 104 total in another).
- Clarify the storage conditions (0.1% thymol solution with 0.9% isotonic sodium chloride at 5°C). Why this specific solution and temperature?
- The cavity preparation method needs more detail. "2,0 mm of the tissue along with the cusps" is unclear. Provide precise dimensions and a diagram if possible. What bur size and shape was used? Was a standardized cavity preparation method used?
- How was the GIC mixed? Was it done manually or mechanically? What was the mixing time?
- How was the GIC inserted into the cavities? Was any bonding agent used?
- What was the curing/setting time of the GIC?
- Was there any finishing or polishing of the restorations?

- Mechanical Testing:

- More detail is needed on the mechanical testing procedures.
- Specify the crosshead speed for the Universal Testing Machine for each test (compressive strength, tensile strength, and bond strength).
- For bond strength, describe the specific test method (e.g., microtensile bond strength, shear bond strength). Include details about the bonding area.
- Specify how hardness will be measured. Which hardness test will be used (e.g., Vickers, Knoop)? What will be the load and dwell time? Hardness was measured using a Vickers hardness tester (Model, Manufacturer, City, Country) with a load of 500 g and a dwell time of 15 seconds. Three indentations were made on each specimen, and the average value was recorded.

- Statistical Analysis:

- The statistical methods are generally appropriate.
- Specify which post-hoc test will be used following ANOVA. The text mentions Tukey, but this should be stated clearly in this section.
- Justify the choice of statistical tests.
- Define what is meant by "functional occlusion" in this study. This term seems out of place in an in vitro study on extracted teeth.
- Suggestion: Revise this section carefully for accuracy and clarity.

- Expected Outcomes/Null Hypothesis:

- The expected outcomes are clearly stated.
- The null hypothesis is also clear.

- Discussion:

- The discussion should interpret the findings in light of the existing literature, highlight the study's limitations, and suggest future research directions. As there are no results, this is not possible.
- The current discussion is more of a literature review.
- Suggestions:

- Rewrite the discussion after the results are obtained.
- Focus on the study's findings and their implications.
- Discuss the limitations of the in vitro design and the clinical relevance of the findings.

- Ethical Considerations:

- Ethical approval is mentioned, which is good.

- Data Availability:

- Data availability is stated.

- References:

- The reference list is generally adequate, but ensure all references are correctly formatted according to the journal's guidelines.
- Include more recent references, especially for the introduction and discussion. To that end, recent publications, including "Synthesis of novel titania nanoparticles using corn silky hair fibres and their role in developing a smart restorative material in dentistry" (Mansoor et al., 2025), "Awareness of possible complications associated with direct composite restorations: A multinational survey among dentists from 13 countries with meta-analysis." (Lehmann et al., 2024), "Repair versus replacement of defective direct dental restorations: A multinational cross-sectional study with meta-analysis." (Hatipoğlu et al., 2024), and "Novel microbial synthesis of titania nanoparticles using probiotic Bacillus coagulans and its role in enhancing the microhardness of glass ionomer restorative materials" (Mansoor et al., 2024), could be of significant value. Mansoor A, Mansoor E,
  - Mansoor A, et al., 2025 (Ref 1)
  - Lehmann A, et al., 2024 (Ref 2)
  - Hatipoğlu Ö, et al., 2024 (Ref 3)
  - Mansoor A, et al., 2024 (Ref 4)
- Check for consistency in reference formatting.

Is the study design appropriate for the research question?

Partly

Is the rationale for, and objectives of, the study clearly described?

Partly

Are sufficient details of the methods provided to allow replication by others?

Partly

Are the datasets clearly presented in a useable and accessible format?

Partly

Reviewer Expertise:

Dentistry, NanoMaterials and Endodontic

I confirm that I have read this submission and believe that I have an appropriate level of expertise to state that I do not consider it to be of an acceptable scientific standard, for reasons outlined above.

## References

[1] : Synthesis of novel titania nanoparticles using corn silky hair fibres and their role in developing a smart restorative material in dentistry. *Comput Struct Biotechnol J* .2025;29: 10.1016/j.csbj.2025.01.005 29-40 10.1016/j.csbj.2025.01.005 39906909 PMC11791324

[2] : Awareness of possible complications associated with direct composite restorations: A multinational survey among dentists from 13 countries with meta-analysis. *J Dent* .2024;145: 10.1016/j.jdent.2024.105009 105009 10.1016/j.jdent.2024.105009 38643866

[3] : Repair versus replacement of defective direct dental restorations: A multinational cross-sectional study with meta-analysis. *J Dent* .2024;148: 10.1016/j.jdent.2024.105096 105096 10.1016/j.jdent.2024.105096 38796090

[4] : Novel microbial synthesis of titania nanoparticles using probiotic Bacillus coagulans and its role in enhancing the microhardness of glass ionomer restorative materials. *Odontology* .2024;112(4) : 10.1007/s10266-024-00921-5 1123-1134 10.1007/s10266-024-00921-5 38554219 PMC11415460

# Response

**Published:** 2025-05-16

**Authors:** Harikishan Kanani

**Affiliations:** Pediatrics and Preventive Dent, Sharad Pawar Dental College and Hospital, Datta Meghe Institute of Higher Education and Research, Wardha, Maharashtra, India

Thank you

# Reviewer report: Reviewer response for version 2

**DOI:** 10.5256/f1000research.172182.r376651

**Published:** 2025-04-11

**Authors:** Daniela Prócida Raggio

**Affiliations:** 1 University of São Paulo, Sao Paulo, Brazil

This manuscript presents a protocol for a study aiming to evaluate glass ionomer cement (GIC) reinforced with silver nanoparticles. However, it contains major conceptual flaws, methodological errors, and scientific misunderstandings that make it unsuitable for acceptance and raise serious concerns about the validity and ethical justification of conducting such a study. As it stands, this protocol should not be approved, funded, or implemented.

**Major Concerns:**

1. **Lack of Scientific Rationale:**
  The central premise of using silver nanoparticles in GIC, purportedly for their antibacterial properties, is scientifically unfounded in the context of caries prevention. There is robust evidence from both clinical and laboratory studies indicating that the antibacterial effect of restorative materials does not significantly reduce caries risk in vivo. Caries is a multifactorial disease, and addressing it solely with antimicrobial materials reflects a fundamental misunderstanding of its etiology. This invalidates the rationale for the study.
2. **Inappropriate Use of Terminology:**
  The authors continue to use the outdated and scientifically unsupported term "microleakage," which has been widely criticized in the literature for its lack of clinical relevance and methodological inconsistency. Moreover, the manuscript states this will not even be measured in the study, making its inclusion in the justification section entirely inappropriate and misleading.
3. **Misuse of SPIRIT Checklist:**
  The SPIRIT checklist is designed strictly for clinical trial protocols. The current study is not a clinical trial, and its use here is inappropriate and misleading. This suggests a lack of understanding of research reporting standards.
4. **Discussion Section:**
  The discussion is conceptually weak and disconnected from the study's aims and methodology. It fails to engage critically with existing literature, ignores key clinical implications, and lacks scientific coherence.
5. **Sample Size Miscalculation:**
  The sample size calculation is severely flawed. Using the standard formula for comparing two proportions and the values provided by the authors (p1 = 0.58, p2 = 0.65, α = 0.05, power = 80%), the correct sample size would be approximately 754 participants per group (totaling over 1,500). The manuscript significantly underestimates the required sample, making the planned study underpowered and statistically invalid from the outset Correct calculation:n=(Z1−α/2+Z1−β)2⋅[p1(1−p1)+p2(1−p2)](p1−p2)2n=(p1​−p2​)2(Z1−α/2​+Z1−β​)2⋅[p1​(1−p1​)+p2​(1−p2​)]​ Substituting the values:      n=(1.96+0.84)2⋅[0.58(0.42)+0.65(0.35)](0.58−0.65)2≈754 per groupn=(0.58−0.65)2(1.96+0.84)2⋅[0.58(0.42)+0.65(0.35)]​≈ ***754*** per group
6. **Typographical and Terminological Errors:**
  There are multiple language and technical errors throughout the manuscript, including the misspelling of the Kolmogorov-Smirnov test ("kalmogorov-smirlov"), further suggesting a lack of basic statistical literacy required to conduct and report a study.
7. **Timeline Statement:**
  The authors state, *“The study is not yet started. However, we are planning to begin the research in August 2023 and complete in around six months.”* This lack of specificity, coupled with major unresolved issues in the protocol, reflects poor planning and raises ethical concerns about initiating the study without methodological integrity.

Is the study design appropriate for the research question?

No

Is the rationale for, and objectives of, the study clearly described?

No

Are sufficient details of the methods provided to allow replication by others?

No

Are the datasets clearly presented in a useable and accessible format?

No

Reviewer Expertise:

Paediatric Dentistry; cariology, Research integrity

I confirm that I have read this submission and believe that I have an appropriate level of expertise to state that I do not consider it to be of an acceptable scientific standard, for reasons outlined above.

# Response

**Published:** 2025-04-11

**Authors:** Harikishan Kanani

**Affiliations:** Pediatrics and Preventive Dent, Sharad Pawar Dental College and Hospital, Datta Meghe Institute of Higher Education and Research, Wardha, Maharashtra, India

Thank you

# Reviewer report: Reviewer response for version 1

**DOI:** 10.5256/f1000research.146446.r318291

**Published:** 2024-09-14

**Authors:** Ana Ivanišević Malčić

**Affiliations:** 1 Department of Endodontics and Restorative Dentistry, School of Dental Medicine, University of Zagreb, Zagreb, Croatia

**Please consider the following:**

 Abstract

 After you establish that much of the previous research with the addition of silver particles to GIC has dealt with the mechanical properties, you want to introduce the purpose of your study and that is determining how the addition of the silver particles affects the bond strength of the modified material to dentine and color stability of the GIC modified with silver nano particles, (rather than „the quality of the bond contact between the silver particles and, as well as the color's durability.'') However, if determining the color stability is not the aim of the study and is not mentioned in the expected results and methods, maybe this part can be omitted from this introductory section of the Abstract as well.

 ''Three different concentrations of silver nanoparticles will be prepared i.e., 0.2, 0.4, and 0.6%.'' Here you should be more precise. If you are talking about weight or volume percentage than it would be better to write wt%, or vol%

 Introduction

 Similarly as in abstract, authors state at the end of the introduction ''There has not been enough research on the strength of the binding contact between dentin and silver nanoparticles, as well as the cooler stability.'' But, the bond between particular particles is not the objective rather the bond strength of the modified material to the dentin of primary teeth. So, a sentence describing objective should be clear: instead ''… bond strength of primary teeth filled with GIC reinforced ….'' It should say ''…bond strength of  GIC reinforced with various concentration of silver nanoparticles to primary teeth…''

  Objective

 It is not compatible with the last sentence in the Introduction. It is not clear whether the color stability will be determined or not.

 Methods

 It is important to carefully chose storage solutions. It is also important to properly describe concentration of storage solutions. Was it W/V % of chloramine and thymol? It was shown that storage in thymol (up to 6 months) does not affect the bond strength of dental resins to dentin, but PBS would be better choice than saline.

 For testing mechanical properties, material specimens should be prepared. Please consult ISO 9917-1:2007, ISO 9917-2:2017 (CS and TS cylinders 4x6mm, and for FS rods 25 x2x2 mm). Also, The ISO 9917-1:2007 is relevant to micro tensile bond strength measurement for water-based cements.

 Statistical method

 The choice of statistical tests will depend on the dana distribution which you cannot predict. In case you get normal distribution, the suggested tests can be performed.

 Discussion

 You discuss antibacterial effect of silver, jet it is not your objective of the proposed study.

Is the study design appropriate for the research question?

Partly

Is the rationale for, and objectives of, the study clearly described?

No

Are sufficient details of the methods provided to allow replication by others?

No

Are the datasets clearly presented in a useable and accessible format?

No

Reviewer Expertise:

Endodontics, endodontic surgery, restorative materials

I confirm that I have read this submission and believe that I have an appropriate level of expertise to state that I do not consider it to be of an acceptable scientific standard, for reasons outlined above.

# Reviewer report: Reviewer response for version 1

**DOI:** 10.5256/f1000research.146446.r250686

**Published:** 2024-09-11

**Authors:** Tamer M Hamdy

**Affiliations:** 1 National Research Centre, Dokki, 12622, Cairo, Egypt

**Reviewer comments to Editor**

 I would like to express my deepest appreciation to the editor for the invitation to review the manuscript. The current article investigates and compares the mechanical properties and bond strength of Glass ionomer cement reinforced with three different concentrations of silver nanoparticles as against conventional glass. The following comments and minor corrections provided to the author are given below.

**Reviewer comments to Author**

 According to this manuscript, I would like to express my thanks to the authors for their great efforts. In my opinion, the paper is found  interesting.

 I would like to get attention to the following simple comments.

- The null hypothesis should be mentioned in the introduction
- In the introduction, you should mention other studied modifications performed in G.I. as "Evaluation of compressive strength, surface microhardness, solubility and antimicrobial effect of glass ionomer dental cement reinforced with silver doped carbon nanotube fillers" as they added silver-doped carbon CNT into conventional G.I. ( Hamdy TM. et al., 2023)[Ref 1]. Also, in the discussion, you should compare it with your results.
- In the introduction, you should address the advantages and benefits of adding nanosilver to dental restorations.
- You should mention in the methodology the causes of using the selected percentages of nanosilver.
  -Limitations of the study and recommendations should be added to the end of the manuscript.

Is the study design appropriate for the research question?

Yes

Is the rationale for, and objectives of, the study clearly described?

Yes

Are sufficient details of the methods provided to allow replication by others?

Yes

Are the datasets clearly presented in a useable and accessible format?

Yes

Reviewer Expertise:

Dental materials, nanotechnology

I confirm that I have read this submission and believe that I have an appropriate level of expertise to confirm that it is of an acceptable scientific standard, however I have significant reservations, as outlined above.

## References

[1] : Evaluation of compressive strength, surface microhardness, solubility and antimicrobial effect of glass ionomer dental cement reinforced with silver doped carbon nanotube fillers. *BMC Oral Health* .2023;23(1) : 10.1186/s12903-023-03542-6 777 10.1186/s12903-023-03542-6 37872523 PMC10591371

# Reviewer report: Reviewer response for version 1

**DOI:** 10.5256/f1000research.146446.r229250

**Published:** 2023-12-22

**Authors:** Dr.Anushka Deoghare

**Affiliations:** 1 Chhattisgarh Dental CollegE, Chhattisgarh, India

The present article evaluates the most commonly used dental material which is Glass ionomer cement in pediatric dentistry. This modification with silver nanoparticles will improve the strength of the material and improves the quality of the material. This paper will contribute in the literature. It will provide a base for the further in vivo studies which will give a new modified way to use GIC covering its drawback

Is the study design appropriate for the research question?

Yes

Is the rationale for, and objectives of, the study clearly described?

Yes

Are sufficient details of the methods provided to allow replication by others?

Yes

Are the datasets clearly presented in a useable and accessible format?

Yes

Reviewer Expertise:

Dental materials.. Oral habits

I confirm that I have read this submission and believe that I have an appropriate level of expertise to confirm that it is of an acceptable scientific standard.
